# Effects of gender-affirming hormone therapy from adolescence to adulthood on cardiovascular function: a cross-sectional study

**DOI:** 10.3389/fendo.2026.1793245

**Published:** 2026-03-18

**Authors:** Silvia Ciancia, Simon D’hulst, Jeroen Vervalcke, Daniel Klink, Guy T’Sjoen, Katya De Groote, Martine Cools, Laura Muiño Mosquera

**Affiliations:** 1 Department of Internal Medicine and Pediatrics, Ghent University, Ghent, Belgium; 2 Pediatric Endocrinology Service, Department of Pediatrics, Ghent University Hospital, Ghent, Belgium; 3 Pediatric Cardiology Service, Department of Pediatrics, Ghent University Hospital, Ghent, Belgium; 4 Department of Endocrinology, Ghent University Hospital, Ghent, Belgium; 5 Department of Endocrinology & Center for Sexology and Gender, Ghent University Hospital, Ghent, Belgium

**Keywords:** aortic distensibility, aortic stiffness, cardiovascular, GAHT, transgender

## Abstract

**Aim:**

Increased cardiovascular risk in transgender adults might be linked in part to metabolic changes associated with gender-affirming hormone therapy (GAHT) and lifestyle factors. However, the cardiovascular effects of long-term GAHT, particularly when initiated during adolescence, remain poorly understood.

**Methods:**

Echocardiographic evaluations were performed in 47 trans men (TM) and 6 trans women (TW) who had undergone GAHT for 5–10 years. Assessments included systolic and diastolic function, ventricular and aortic diameters, and aortic elasticity parameters. Cardiovascular risk factors (e.g., hypertension, obesity, impaired glucose tolerance, dyslipidemia, smoking, and alcohol use) were also analyzed.

**Results:**

Median (IQR) GAHT duration was 6.0 (2.8) years in TM and 7.8 (2.6) years in TW; median ages were 23.4 (2.2) and 25.3 (2.7) years, respectively. Most of the cardiovascular parameters were within normal range, as all participants showed normal systolic function, and only one TM exhibited grade 2 diastolic dysfunction. Additionally, left ventricle (LV) diameters, LV mass indexed for BSA and aortic diameters were also within normal reference ranges in both groups. Nevertheless, GAHT was associated with significant reduction of aortic distensibility and strain in comparison to normal reference values, while increasing aortic stiffness index in both TM and TW. In TM, reduced aortic distensibility was independently associated with increases in systolic blood pressure.

**Conclusions:**

Long-term GAHT initiated during adolescence in both TW and TM shows no apparent cardiac complications with regard to cardiac function, hypertrophy, or chamber dimensions, and does not significantly affect aortic diameters. However, alterations in aortic elasticity were observed, the long-term clinical significance of which remains to be determined.

## 1 Introduction

Transgender and gender diverse (TGD) individuals do not identify with the sex registered at birth (SRAB), experiencing gender incongruence that can lead to intense psychological distress known as gender dysphoria. To align physical characteristics with their experienced gender (EG), many TGD people seek support from gender clinics for psychological care and gender-affirming medical and/or surgical treatments. However, not all TGD persons choose to undergo medical treatment or surgery (1–3).

**Figure 1:**
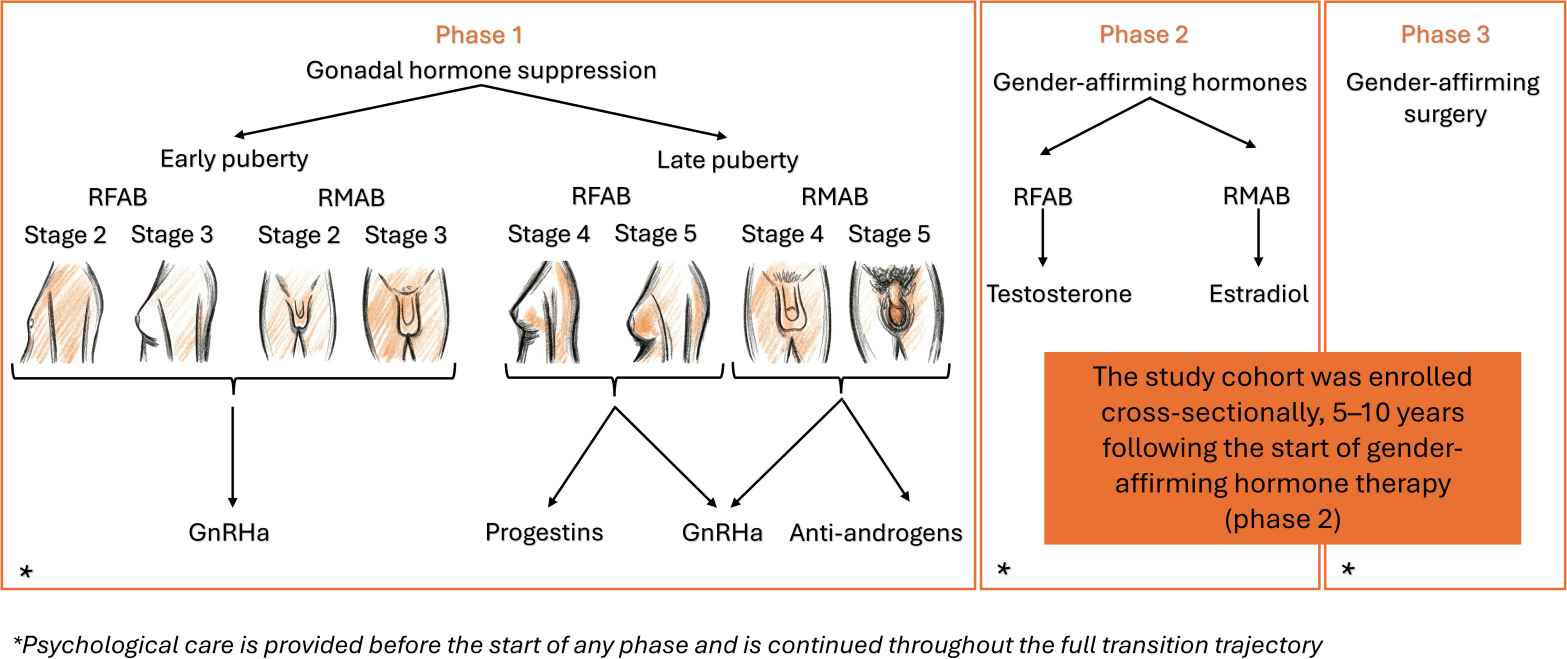
Gender-affirming trajectory in transgender adolescents.

In the last decades, the number of minors presenting to gender clinics and pursuing medical gender-affirmation has drastically increased (4–7). Gender-affirming medical treatment during adolescence typically involves two phases: suppression of sex steroids (testosterone, estradiol) that are naturally produced from puberty onwards (gonadal hormone suppression, GHS) followed, if desired, by gender-affirming hormone therapy (GAHT). GHS can be achieved using gonadotropin-releasing hormone agonists (GnRHa) from the onset of puberty onwards. In later stages of puberty, alternative medications such as progestins in individuals registered female at birth (RFAB) or anti-androgens in individuals registered male at birth (RMAB) may also be used. GAHT can then be started, with testosterone (usually intramuscular or transdermal) for RFAB individuals and estradiol (usually oral or transdermal) for RMAB individuals. Gender-affirming surgery is generally reserved for adults, although mastectomy may be considered in selected cases before the age of 18 (1, 3), see **Figure 1**.

Studies in adult populations suggest that TGD individuals may have a higher risk of cardiovascular events (8–12). While the causes remain unclear, metabolic changes linked to GAHT and lifestyle factors may contribute (13). Feminizing GAHT may increase thromboembolic risk due to pro-thrombotic effects of estrogens, including elevated pro-coagulation and pro-inflammatory factors (12, 14–16). It is also associated with increased fat mass, reduced lean mass, and potentially higher insulin resistance (13, 17). However, feminizing GAHT may improve lipid profiles by lowering LDL cholesterol (LDL-C) and raising HDL cholesterol (HDL-C) (13). Masculinizing GAHT may increase cardiovascular risk by raising hematocrit and red blood cell counts, elevating blood pressure, reducing HDL-C, and increasing LDL-C (13, 18). Conversely, it often leads to greater lean mass and reduced fat mass, which may enhance insulin sensitivity (17, 19). Cardiovascular risk in TGD individuals may therefore shift toward that of their affirmed gender (AG), highlighting the importance of understanding these changes to guide counseling and care. Moreover, those who begin GAHT in adolescence are exposed to synthetic hormones during a developmental period when cardiovascular sexual dimorphism is still forming. Although less apparent than in other organs, sex-based differences exist in cardiac structure and physiology; for example men typically have greater ventricular mass and aortic and arterial size, while women have higher heart rates and higher ejection fraction (20). While much of this dimorphism emerges at puberty and is attributed to hormonal effects, it is often difficult to separate hormonal influences from genetic factors (21).

Therefore, investigating the impact of GAHT initiated during puberty on cardiac dimension, function, and cardiovascular risk factors is essential for informed counseling and protection of long-term cardiovascular health in TGD individuals. In this study, we evaluated cardiac and aortic diameters and function, and cardiovascular risk factors in a population of TGD young adults who began GAHT during adolescence. Because the cohort of trans men (TM) was substantially larger than the cohort of trans women (TW), the results concerning TW must be interpreted cautiously. Even so, they offer important preliminary insights.

## 2 Methods

### 2.1 Study design and objectives

This cross-sectional study was approved by the Ethics Committee of Ghent University Hospital (reference: BC-06676) and conducted in accordance with the principles of the Declaration of Helsinki. All participants (and legal guardians if appropriate) provided written informed consent prior to participation. Recruitment occurred between September 2023 and December 2024. The primary focus of the study was the assessment of cardiovascular function, specifically systolic and diastolic function, as well as the presence of ventricular hypertrophy and aortopathy. The secondary aim was to identify potential risk factors that could explain abnormal echocardiographic findings.

### 2.2 Study population

All individuals who had initiated medical gender-affirmation during adolescence within the Pediatric Gender Team at Ghent University Hospital and who had been receiving GAHT for a minimum of five years and a maximum of ten years at the time of the study visit were invited to participate in a comprehensive cross-sectional study, aiming to evaluate bone health, body composition, muscle strength, voice changes and cardiovascular function (22, 23).

All assessments were conducted on the day of the participants’ regular consultation at the Gender Service, except for the echocardiogram, which was scheduled on a separate day. Out of 231 eligible individuals, 117 (91 TM, 26 TW) consented to participate in the study. Among them, 53 participants (47 TM, 6 TW) also agreed to undergo cardiac evaluation. A comparison of characteristics at enrollment between those who completed the cardiac assessment and those who declined it is presented in **Supplementary Table 1**. In addition, an exploratory cohort of transgender adolescents (n=18; 12 RFAB, 6 RMAB), prior to initiation of medical treatment, was included to compare aortic elasticity parameters with published normative data (24).

Of note, during the medical gender-affirmation process, participants were treated according to standard protocols. At our center, until September 2022, GnRHa were prescribed to achieve GHS only to early pubertal adolescents (Tanner stage 2–3), because of reimbursement criteria. Late pubertal adolescents (Tanner stage 4-5) received progestins (RFAB) or anti-androgens (RMAB). GAHT was initiated following standard protocols: testosterone esters for RFAB (starting with 50 mg i.m. every 2 weeks) and estradiol valerate for RMAB (0.5 mg/day, orally), with doses increased every 6 months up to the adult replacement dose.

### 2.3 Study procedures

A fasting blood draw was performed to determine sex hormone levels and glucolipid profile. If fasting was not confirmed at the time of sampling, this was documented. Physical activity was assessed using an accelerometer (ActiGraph wGT3X-BT), a validated wearable device for academic research that captures movement and estimates activity levels. Participants wore the device continuously for seven consecutive days, including during sleep. The only exceptions were water-based activities such as bathing, showering, or swimming. If swimming was the participant’s regular sport, this was recorded to account for potential underestimation of physically active time. Participants also completed a questionnaire covering education, employment, smoking, alcohol use, and recreational drug use. Blood pressure was measured three times, and the average of the readings was used for analysis. Lastly, an echocardiogram was performed.

### 2.4 Definition of cardiovascular risk factors

Cardiovascular risk factors were defined based on the most recent guidelines (25–31), as summarized in **Supplementary Table 2**. Lipid profiles were assessed regardless of fasting status at the moment of the sampling, in line with the guidelines from the European Society of Cardiology (ESC) and supporting meta-analysis (27, 32). When a different cut-off for men and women was indicated (i.e. waist circumference, HDL-C, drinking risk), TGD individuals were primarily classified by the AG.

### 2.5 Echocardiographic assessment

Echocardiographies were performed with a Vivid™ E95 or Vivid™ S70N, GE Healthcare, equipped with a M5Sc probe. Data were stored and analyzed offline using an ultrasound workstation (EchoPAC, GE Healthcare, version 206). For continuous variables, an average of ≥3 consecutive measurements was calculated. Left ventricular dimensions and function were measured from M-mode images in parasternal short-axis view. Left ventricular ejection fraction (LVEF) was calculated according to the Teicholz-method (LVEF = (EDV-ESV)/EDV, where EDV/ESV = [7/(2.4+LVIDd/s)]*LVIDd/s^3^; EDV, end diastolic volume; ESV, end-systolic volume; LVIDd/s, left ventricular internal diameter in diastole/systole) and fractional shortening (FS) was calculated as FS = [(LVIDd-LVIDs)/LVIDd]*100. Left ventricular (LV) mass was calculated as follows: LV mass = 0.8*1.04*[(IVSd+LVIDd+LVPWd)^3^-LVEDd^3^]+0.6g (IVSd, interventricular septal end diastole; LVPWd, left ventricular posterior wall in end diastole) (33). Both ventricular dimensions and LV mass were interpreted according to current reference values (33). These were categorized by SRAB and EG into normal, mildly, moderately, or severely abnormal (34). Diastolic LV function was assessed using both pulsed-wave and tissue doppler imaging. Right ventricular function was assessed through tricuspid annular plan systolic excursion (TAPSE) on apical four chamber view in M-mode. Aortic diameters were measured according to the guidelines of the American Society of Echocardiography (ASE) and the European Association of Cardiovascular Imaging (EACVI) (33). Aortic root and ascending aortic z-scores were calculated according to Campens et al. (35). Aortic elasticity parameters, such as distensibility, strain, and stiffness index, were measured from M-mode images of the ascending aorta at the level of the right pulmonary artery. Aortic distensibility was calculated according to Elçioğlu et al. and compared with their reference values, derived using the same M-mode methodology in a healthy, age-stratified cohort (aortic distensibility = 2* [(Ao_s_-Ao_d_)/Ao_d_]/(SBP-DBP)), where Ao_s/d_, aortic diameter in systole/diastole; SBP, systolic blood pressure; DBP, diastolic blood pressure (36). Aortic strain and stiffness index were calculated as follows: aortic strain = 100*(Ao_s_-Ao_d_)/Ao_d_; aortic stiffness index = ln(SBP/DBP)/[(Ao_s_-Ao_d_)/Ao_d_] (36). Measurements in the pre-treatment cohort were compared with published age-adjusted reference values for healthy adolescents (24).

In the absence of a control group, the use of reference values for all cardiac and aortic measurements provided a standardized framework for interpretation, accounted for growth-related variability, while also enabling reproducibility and comparability across studies.

Sex-dependent cardiac and aortic parameters were categorized for both SRAB and AG to assess whether GAHT shifted these measurements toward the typical range for the AG.Observer variability was assessed in a subset of 10 random participants. Two independent, blinded observers each performed four repeated M-mode measurements of ascending aortic systolic and diastolic diameters. Intraobserver reliability was excellent for both diastolic (ICC(2,1) = 0.95 and 0.98) and systolic measurements (ICC(2,1) = 0.96 and 0.99). Interobserver agreement was excellent for diastolic (ICC(2,1) = 0.90) and good for systolic diameters (ICC(2,1) = 0.80).

### 2.6 Statistical analysis

Descriptive statistics were used to summarize the characteristics of the study population. Categorical variables are presented as frequencies and percentages. Continuous variables are reported as means with standard deviations (± SD) for normally distributed data, or as medians with interquartile ranges (IQR) for non-normally distributed data. Intra- and interobserver reliability was evaluated by two-way random-effects intraclass correlation coefficients (ICC(2,1)). Correlation analysis was performed to explore the relationship between left ventricular mass indexed to body surface area (BSA) and the duration of GAHT. Multivariable linear regression models were applied to explore associations between aortic distensibility, stiffness index, and strain, and covariates selected *a priori* based on clinical relevance (systolic blood pressure, BMI, fasting glucose levels, LDL-C, and GAHT duration).Model assumptions (linearity, homoscedasticity, normality of residuals, and multicollinearity) were assessed, and models were kept parsimonious to avoid overfitting. One-way ANOVA was used to evaluate differences in these three vascular outcomes according to smoking status (never smoker, former smoker, current smoker). Depending on the distribution of the data, either the independent samples t-test or the Wilcoxon rank-sum test was used to assess differences based on drinker/non-drinker status. One sample t-tests were used to compare normally distributed aortic elasticity parameters with published reference values, and one-sample Wilcoxon signed-rank tests were applied for non-normally distributed variables. Statistical significance was defined as a two-sided p-value < 0.05. All analyses were conducted using RStudio, version 4.4.3.

## 3 Results

### 3.1 Characteristics at enrollment

A total of 53 participants were enrolled, of whom 47 TM and 6 TW. Mean age at the enrollment was 23.6 (± 1.5) years in TM and 25.3 (± 3.1) in TW. Median age at the start of GHS was 16.4 (1.5) years in TM and 16.5 (1.6) in TW. Median age at the start of GAHT was 17.1 (1.5) years in both groups. The median duration of GAHT at the time of the study was 6.0 (2.8) years in TM, and 7.8 (2.6) years in TW.

At the start of GHS, 46 TM were post-menarche (Tanner stage 4-5) and had received progestins while one had started GHS at Tanner stage 2 with GnRHa. At enrollment 3 participants were still taking progestins, while 44 had stopped GHS. With regard to GAHT, 41 were using testosterone esters (intramuscular), 5 testosterone undecanoate (intramuscular) and 1 was using testosterone gel. None of the TM participants was receiving cardiovascular medications or had had previous cardiovascular events. Among TW, 5 were late pubertal (Tanner stage 4-5) at the start of GHS and received cyproterone acetate (CPA), while 1 was Tanner stage 3 and received GnRHa. At enrollment, only one TW participant was still taking CPA. Five out of six were taking estradiol valerate, one was using estradiol gel. One TW participant was taking bisoprolol at the enrollment, because of sinus tachycardia.

The hormonal profile for both TM and TW is shown in **Table 1**. Cardiovascular risk factors are summarized in **Table 2**.

**Table 1:** Hormonal profile in trans men and trans women at the enrollment.

Endocrine marker	Trans men (n=47)	Trans women (n=6)
FSH (M: 1.5 – 12.4 U/L F: 1.7-21.5 U/L, depending on menstrual phase)	2.0 (6.4)	31.5 (33.8)
LH (M: 1.7 – 8.6 U/L F: 2–96 U/L, depending on menstrual phase)	1.45 (5.5)	30.0 (24.0)
Estradiol (M: 8.0-42.0 ng/L F: 50–400 ng/L, depending on menstrual phase)	33.0 (23.5)	68.0 (6.0)
Total testosterone (M: 267–929 ng/dL F: 11–59 ng/dL)	380.4 (399.0)	16.7 (10.3)
Free testosterone (M: 6–25 ng/dL F: 0.02-0.64 ng/dL)	7.8 (5.4)	0.0 (0.2)
SHBG (M: 11.6-71.2 nmol/L F: 10.5-163.7 nmol/L)	22.9 (13.2)	68.7 (57.6)

**Table 2:** Cardiovascular risk factors.

Risk factor	Trans men (n=47)	Trans women (n=6)
Blood pressure (BP)		
Mean Systolic BP (mmHg ± SD)	116 ± 9	112 ± 8
Mean Diastolic BP (mmHg ± SD)	70 ± 6	69 ± 6
Hypertension	0	0
Elevated BP (max value)	13 (27.7%) (133/84 mmHg)	1 (16.7%) (120/82 mmHg)
Obesity		
Obesity (BMI-based)		
Class I (BMI 30–34.9)	4 (8.5%)	1 (16.7%)
Class II (BMI 35–39.9)	3 (6.3%)	0
Class III (BMI ≥40)	2 (4.3%)	0
Waist circumference		
>102 cm	5 (10.6%)	0
>88 cm	14 (29.8%)	1* (16.7%)
Lipid profile		
Triglycerides >150 mg/dL (max value)	9 (19.1%) (389)	0
Total-C >190 mg/dL (max value)	14 (29.8%) (281)	2 (33.3%) (208)
LDL-C >116 mg/dL (max value)	13 (27.7%) (183)	1 (16.7%) (170)
LDL-C >100 mg/dL	25 (53.2%)	2 (33.3%)
HDL-C below recommended for AG	15 (31.9%)	1 (16.7%)
Glucose		
Fasting Glucose >100 mg/dL (max value)	4 (8.5%)** (120)	0
Smoking status		
Never smoker	28 (59.6%)	1 (16.7%)
Former smoker	7 (14.9%)	4 (66.7%)
Current smoker	11 (23.4%)	1 (16.7%)
Alcohol consumption		
Regular drinkers	16 (34%)	3 (50%)
Low-risk drinkers	14 (29.8%)	2 (33.3%)
Moderate-risk drinkers	1 (2.1%)	0
Data non available	1 (2.1%)	1 (16.7%)
Physical activity		
<150 min/week (Insufficient)	2 (4.3%)	1 (16.7%)
150–300 min/week (Recommended)	8 (17%)	1 (16.7%)
>300 min/week (High)	31 (66%)	2 (66.7%)
Data not available	6 (12.8%)	2 (66.7%)

*The waist circumference for this participant was 102 cm. **All four participants were not fasting or had unknown fasting status at the blood sampling.

### 3.2 Ventricular systolic and diastolic function

An overview of all systolic and diastolic functional parameters is given in **Table 3**. In both TM and TW, systolic function was normal in all participants, as reflected by normal TAPSE, fractional shortening and ejection fraction. With regard to diastolic function, in both groups early diastolic mitral inflow velocity (E wave) and early diastolic mitral annular velocities (e′) at the lateral and septal walls were mildly elevated, although this is often observed in healthy young adults. The mean E/A ratio exceeded 1 in both groups, indicating normal diastolic filling patterns. The mean E/e′ ratio remained within normal limits across participants, suggesting the absence of elevated filling pressures. Comprehensive analysis of these parameters identified grade II diastolic dysfunction only in one TM. This participant commenced GHS with progestins at 15.9 years, followed by testosterone at 16.2 years. At the enrollment (age 21.2 years), he was receiving intramuscular testosterone esters (125 mg every 2 weeks). He did not report comorbidities or concurrent medications, except class I obesity (BMI 31.6 kg/m²). Laboratory tests revealed mild hyperlipidemia (triglycerides 389 mg/dL, total-C 205 mg/dL and LDL-C 111 mg/dL); however, fasting status at the time of sampling was not documented. He abstained from alcohol and was a former smoker, and practiced regular physical activity (>300 min/week).

**Table 3:** Echocardiographic assessment of systolic and distolic function, cardiac morphology and aortic parameters.

Parameter	Trans men	Trans women	References
Systolic function			
TAPSE (mm)	22.5 (± 3.4)	23.6 (± 4.0)	≥17
Fractional shortening (%)	34.1 (± 3.9)	36.6 (± 3.5)	>25
Ejection fraction (%)	62.8 (± 5.1)	66.0 (± 4.0)	Males: 52-72 Females: 54-74
Diastolic function			
MV E velocity (cm/s)*	81.2 (± 17.4)	86.7 (± 20.9)	Males: 52-111 Females: 59-122
MV e’ lateral velocity (cm/s)*	15.5 (± 3.7)	15.8 (± 4.2)	Males: 9.09-21.8 Females: 10.83-23.31
MV e’ septal velocity (cm/s)*	11.2 (± 2.2)	13.9 (± 2.5)	Males: 7.02-15.87 Females: 8.21-17.37
E/A ratio*	1.6 (± 0.4)	1.8 (± 0.14)	Males: 0.9-2.7 Females: 1.07-2.99
E/e’ ratio*	6.4 (± 1.4)	5.9 (± 0.5)	Males: 4.06-9.13 Females: 4.47-10.01
Cardiac dimension			
IVSd (mm)	8.8 (± 1.4)	8.1 (± 0.9)	Males: 6-10 Females: 6-9
LVIDd (mm)	49.0 (± 4.9)	50.3 (± 4.7)	Males: 42.0-58.4 Females: 37.8-52.2
LVIDd/BSA (cm/m^2^)	2.7 (± 0.3)	2.6 (± 0.2)	Males: 2.2-3.0 Females: 2.3-3.1
LVPWd (mm)	8.4 (± 1.2)	8.2 (± 0.3)	Males: 6-10 Females: 6-9
LV mass/BSA (g/m^2^)	78.3 (± 15.3)	73.9 (± 12.9)	Males: 49-115 Females: 43-95
Aortic indexes			
Aortic sinus diameter (mm)	29.4 (± 2.4)	29.4 (± 2.8)	–
Aortic sinus Z-score AG	-0.3 (± 0.9)	0.4 (± 0.9)	–
Aortic sinus Z-score SRAB	0.6 (± 0.8)	-0.5 (± 0.9)	–
Ascending aorta diameter	25.4 (± 2.9)	24.8 (± 1.9)	–
Ascending aorta Z-score AG	-0.5 (± 0.9)	-0.5 (± 0.8)	–
Ascending aorta Z-score SRAB	-0.04 (± 0.9)	-0.9 (± 0.8)	–
Ascending aorta distensibility (cm^2^/dyn^1^/10^3^)	3.79 (± 1.67)	3.10 (± 0.95)	9.1 (± 1.86)
Ascending aorta strain (%)	11.28 (± 4.56)	8.58 (± 2.28)	18.73 (± 3.13)
Ascending aorta stiffness index	5.21 (± 2.33)	5.94 (± 1.94)	2.65 (± 0.55)

Values are expressed as mean (± SD). *Normal values for diastolic function in a young population (18–40 y) published by Miyoshi et al., 2020 (31). All other references are derived from the work of Lang et al., 2015 (30).

TAPSE, tricuspid annular plane systolic excursion; MV, mitral valve; E/A ratio, the ratio of early (E) to late (A) ventricular filling velocities measured by pulsed-wave Doppler across the mitral valve. E/e’ ratio: the ratio of mitral inflow E velocity (E) to tissue Doppler e’ velocity (from the mitral annulus, mean of lateral and septal). IVS, interventricular septum thickness; LVID, left ventricular internal diameter; LVPW, left ventricular posterior wall; BSA, body surface area; AG, affirmed gender; SRAB, sex registered at birth.

### 3.3 Cardiac dimensions

Echocardiographic assessment of cardiac dimensions revealed that both TM and TW exhibited interventricular septum, LV internal diameter and LV posterior wall thicknesses within normal reference ranges. LV mass indexed for BSA was also within normal ranges in both groups, as shown in **Table 3**.

After correction for BSA, a mildly dilated LV was observed in 6 TM when categorized by AG, but only in 3 when categorized by SRAB. LV mass indexed for BSA was mildly increased in 7 TM when categorized by SRAB, but in none when categorized by AG. No correlation between LV mass/BSA and testosterone therapy duration was found (Pearson’s coefficient = -0.03). In TW, no dilation or LV mass increase was detected, neither for AG or SRAB.

### 3.4 Aortic diameters, stiffness and distensibility

In TM, the Z-scores of the aortic sinus were all below +2 when calculated according to AG, whereas two participants showed values above +2 (max 2.68) when using SRAB as reference. For the ascending aorta, only one TM had Z-scores above +2 (2.9 for AG; 3.1 for SRAB). In TW, Z-scores were consistently below +2 at both sites (sinus and ascending aorta). The comparison between Z-scores based on AG and SRAB revealed a statistically significant difference (p < 0.001), indicating that the aortic sinus diameter aligns more closely with reference values for AG in both TM and TW. At the ascending aorta, Z-scores were more consistent with SRAB in TM, while in TW they were more aligned with AG values, although this may be due to the small sample size of TW. It is also worth noting that, in adults, measurements at the ascending aorta are generally less reliable than those at the sinus, and although a degree of sexual dimorphism exists, aortic diameters do not markedly differ between men and women. Aortic diameter values are shown in **Table 3**.

Aortic distensibility and strain were significantly reduced in both TM and TW compared to reference values (all p<0.001; in TM mean difference -5.31, 95% CI -5.80 to -4.82 for aortic distensibility, and -7.45, 95% CI -8.79 to -6.11 for aortic strain; in TW -6.0, 95% CI -7.0 to -5.0, and -10.15, 95% CI -12.54 to -.7.75, respectively) (36), while the aortic stiffness index was significantly increased (mean difference 2.56, 95% CI 1.88 to 3.25, p<0.001 for TM and mean difference 3.29, 95% CI 1.25 to 5.33, p<0.009 for TW) (**Table 3**).

### 3.5 Risk factors associated with abnormal aortic stiffness

Multivariate regression analysis was performed in TM to assess associations between aortic distensibility, stiffness index, and strain. Systolic blood pressure was independently associated with reduced aortic distensibility after adjustment for BMI, glucose, LDL-C, and duration of GAHT. However, in the absence of a control group, the direct effect of GAHT could not be isolated from other contributing factors. No significant associations emerged between stiffness index or strain and cardiovascular risk factors (**Table 4**). Additionally, group comparisons across smoking status (never, former, current smoker) and alcohol consumption (drinkers vs. non-drinkers) revealed no significant differences. Due to the limited sample size, these analyses were not conducted in TW.

**Table 4:** Multivariate regression analysis of aortic distensibility, stiffness index, and strain index in trans men.

	Aortic distensibility			Aortic stiffness index			Aortic strain index		
Covariate	Estimate	95%CI	p-value	Estimate	95%CI	p-value	Estimate	95%CI	p-value
Systolic BP	-0.08	(-0.14; -0.03)	**0.004**	0.05	(-0.03; 0.14)	0.191	-0.13	(-0.29; 0.03)	0.107
BMI	-0.02	(-0.11; 0.07)	0.631	0.08	(-0.05; 0.21)	0.229	-0.07	(-0.33; 0.19)	0.574
Glucose	0.04	(-0.00; 0.08)	0.070	-0.05	(-0.11; 0.01)	0.131	0.10	(-0.02; 0.22)	0.103
LDL-C	-0.01	(-0.02; 0.01)	0.513	0.01	(-0.02; 0.04)	0.411	-0.03	(-0.08; 0.03)	0.335
GAHT duration	0.02	(-0.28; 0.33)	0.881	-0.09	(-0.56; 0.38)	0.714	0.03	(-0.89; 0.95)	0.950

p-values in bold are significant (<0.05). BP, blood pressure; BMI, body mass index; LDL-C, cholesterol LDL; GAHT, gender-affirming hormone therapy.

### 3.6 Aortic elasticity parameters in the pre-treatment cohort

In the untreated cohort of TGD adolescents (mean age 12.0 ± 1.3 years), aortic distensibility was consistent with age-adjusted reference values (94.96 ± 29.18 vs 97.1 ± 47.60; mean difference −2.14, 95% CI −16.65 to 12.37; p=0.76). The mean stiffness index was significantly higher than the published normative mean (2.28 ± 0.63 vs 1.18 ± 0.57; mean difference 1.10, 95% CI 0.79 to 1.41; p < 0.001). However, all individual values remained within the reported normal reference range (cohort range 1.37-3.45 vs normative range 0.24-3.69), providing no indication of increased aortic stiffness in this cohort.

## 4 Discussion

In this cross-sectional study, we investigated cardiac dimensions, function, and aortic parameters in a cohort of adult TGD individuals who began GAHT during adolescence. To our knowledge, this is one of the few studies evaluating echocardiographic and aortic parameters in TGD individuals several years after initiation of GAHT in puberty, providing insights into long-term cardiovascular effects of early medical gender-affirmation (37). Reassuringly, systolic function was within normal ranges in all participants, while diastolic function was mildly impaired only in one TM, who also had obesity and hyperlipidemia. LV dimensions, including LV mass indexed to BSA, were within normal limits across groups. However, interpretation varied depending on whether SRAB or AG reference values were applied. In fact, in TM mild LV dilation and increased LV mass were more frequently identified when using SRAB references, in line with previous findings (37). Aortic sinus Z-scores calculated based on AG were more consistent with the observed diameters than those calculated based on SRAB. In contrast, Z-scores at the ascending aorta in TM remained more closely aligned with SRAB-based norms, although this may reflect the inherent difficulty in measuring the ascending aorta in adults on echocardiography. On one hand these findings underscore the importance of selecting appropriate reference value in clinical evaluation of TGD individuals, as cardiovascular parameters may align more closely with the AG than with SRAB after long-term GAHT. On the other hand, longer follow-up is needed to determine whether the observed increase in LV mass reflects a physiological shift toward values typical of the AG or the early development of a pathological process leading to LV hypertrophy and potentially adverse cardiac outcomes. Similarly, aortic sinus diameters should be monitored over the long term to exclude progressive aortic root dilation. A notable finding was the significant reduction in aortic distensibility and strain, alongside increased stiffness index, in both TM and TW compared to established normative values. To explore this further, these parameters were also evaluated in a small cohort of transgender adolescents (n = 18) prior to any medical treatment. In contrast to the main cohort, our exploratory analysis showed aortic stiffness parameters largely within aged-adjusted normative ranges (24). As abnormal aortic distensibility and aortic stiffness were not observed in our cohort of pre-treatment TGD individuals, we hypothesize that GAHT, particularly long-term exposure initiated during adolescence, might contribute to early vascular stiffness. The clinical implication of this finding needs further research, as aortic stiffness is an early marker of vascular aging and also an independent predictor of stroke and coronary artery disease across diverse populations (38). In young adults, reduced aortic elasticity may not translate into immediate cardiovascular events but could represent a long-term risk factor, particularly in the presence of additional cardiovascular risk factors such as hypertension or dyslipidemia. In our cohort, multivariable analysis identified an association between systolic blood pressure and aortic distensibility in TM. However, no associations were found with other cardiovascular risk factors or GAHT duration. This is likely due to the relatively small sample size and the homogeneous duration of GAHT within the cohort. Furthermore, the lack of a cisgender control group limits causal inference. Nonetheless, these findings align with previous studies reporting increased arterial stiffness in TM on long-term GAHT (39, 40). Increased arterial stiffness in TW has not been reported earlier (41). Several factors may help explain our observations. Although an increase in aortic distensibility would typically be expected following feminizing GAHT, exogenous estrogens may have less favorable effects on the vascular wall compared to endogenous estrogens. The median estradiol level in our cohort (68 pg/mL) fell slightly below the recommended range of 100–200 pg/mL (1), likely due to the relatively low oral doses used (1–4 mg/day) as well as to individual differences in absorption and metabolism. Also, almost all TW used oral estradiol instead of transdermal formulations, which may be more effective in providing cardiovascular protection (42). As a result, the expected cardioprotective effects of estradiol may not have been fully achieved. Our findings differ from those of Sharula et al. (41), who reported reduced arterial stiffness in TW receiving estrogen therapy. One important distinction between the studies is the timing of GAHT initiation. In our cohort TW initiated GAHT at an earlier age, whereas participants in the study of Sharula et al. began treatment during adulthood. In addition, different estrogen formulations were used across studies. There were also key methodological differences: Sharula et al. compared TW on GAHT to TW not receiving hormone therapy, while we compared our cohort to age-matched reference values from a healthy cisgender population. Furthermore, Sharula et al. conducted their study in Japan, and ethnic variability may also contribute to differences in vascular outcomes. Finally, our TW sample size is small, limiting the reliability of any results. Nevertheless, these findings underscore the need for further research on the vascular effects of feminizing hormone therapy, particularly when initiated during adolescence.

Importantly, early medical gender-affirmation and access to gender-affirming care in young TGD individuals provides an opportunity for timely cardiovascular risk assessment and the implementation of preventive strategies, potentially fostering healthier long-term outcomes. Mental health comorbidities, such as depression, along with experiences of discrimination and reduced social acceptance, can negatively affect health behaviors. These challenges may lead to decreased physical activity and increased engagement in harmful habits such as smoking or alcohol use, further emphasizing the need for early prevention efforts.

This study offers several noteworthy strengths. First, it focuses on a unique and under-researched population, i.e. young transgender adults who initiated GAHT during adolescence, providing valuable insight into the long-term cardiovascular effects of early medical gender-affirmation. The standardized protocol, conducted in a single tertiary center, reduces inter-observer variability and supports the internal validity of the data. The inclusion of both TM and TW allows for a comparative approach and enhances the generalizability of findings across the transgender spectrum. The comprehensive cardiovascular assessment, with echocardiography as the primary modality, is another key strength, as echocardiography is easily available. It enabled detailed evaluation of both cardiac dimensions and function, as well as vascular parameters such as aortic diameters, distensibility, and stiffness, adding depth to the analysis beyond traditional cardiovascular risk factors.

Nevertheless, several limitations must be acknowledged. First, the cross-sectional design precludes conclusions about causality or temporal progression. Second, the sample size of TW was limited, restricting statistical power and generalizability, and making findings in this subgroup exploratory. However, this imbalance is partially a consequence of the current clinical reality among TGD adolescents, for whom the ratio of TM to TW presenting to gender clinics is approximately 3:1, and participation rates in research studies are typically lower among TW than TM. In addition, the stepwise inclusion process (231 eligible patients, 117 participating in the main study, and 53 completing the separate cardiovascular assessment) may have introduced selection bias, as participation in the cardiovascular substudy may have been influenced by logistical burden, level of engagement in care, and perceived relevance of the additional assessment. The absence of a matched cisgender control group limits interpretation of GAHT-specific effects. Finally, fasting status was not confirmed for all blood samples, although this reflects real-world clinical practice and is in line with current ESC guidelines.

Despite these limitations, this study provides valuable insight into the long-term cardiovascular health of TGD individuals initiating GAHT during adolescence. Our findings support the relative cardiac safety of early GAHT but raise important questions about long-term vascular health. Future studies adopting longitudinal designs, with larger, possibly multicenter cohorts, and including cisgender controls are needed to clarify the trajectory and determinants of cardiovascular remodeling in this population. The integration of imaging modalities such as cardiac MRI and MR angiography with pulse wave velocity may provide more comprehensive cardiovascular assessment. In parallel, echocardiographic monitoring of TGD youth receiving GAHT should be incorporated into clinical follow-up (e.g., every five years), particularly given potentially concerning findings such as increased LV mass in TM and elevated aortic stiffness in both TM and TW.

In conclusion, young TGD adults who began GAHT during adolescence exhibited normal cardiac function and dimensions, with structural characteristics more closely aligned with their AG. However, signs of reduced aortic elasticity and increased vascular stiffness were observed, potentially reflecting early vascular changes associated with GAHT. These findings underscore the need for individualized cardiovascular risk assessment and long-term follow-up in TGD populations undergoing medical gender-affirmation during adolescence.

## Data Availability

The raw data supporting the conclusions of this article will be made available by the authors, on reasonable request.
