# Supplementary material for: Effects of gender-affirming hormone therapy from adolescence to adulthood on cardiovascular function: a cross-sectional study
Source: Front Endocrinol (Lausanne). 2026 Mar 18;17:1793245. doi: 10.3389/fendo.2026.1793245 (PMC13038501; doi:10.3389/fendo.2026.1793245)
Supplement: Supplementary file 1 [file Table1.docx]

**Supplementary table 1.** Comparison of baseline characteristics between those who completed the cardiac assessment and those who declined.

|  | **Cohort undergoing cardiac evaluation** | | **Cohort declining cardiac evaluation** | |
| --- | --- | --- | --- | --- |
|  | Trans men  (n=47) | Trans women (n=6) | Trans men  (n=44) | Trans women  (n=20) |
| **Gonadal hormone suppression (GHS)** |  | | | |
| Age at start GHS (years) | 16.4 (1.5) | 16.5 (1.6) | 15.9 (2.5) | 16.0 (3.6) |
| GHS achieved through progestins/CPA | 46 (97.9%) | 5 (83.3%) | 36 (81.8%) | 15 (75%) |
| GHS achieved through GnRHa* | 1 (2.1%) | 1 (16.7%) | 4 (9.01%) | 5 (25%) |
| **Gender-affirming hormone therapy (GAHT)** |  | | | |
| Age at start GAHT (years) | 17.1 (1.5) | 17.1 (1.5) | 17.3 (1.8) | 16.9 (2.2) |
| **Enrollment** |  | | | |
| Age at the enrollment (years) | 23.6 (±1.5) | 25.3 (±3.1) | 23.6 (±1.6) | 24.0 (±1.6) |
| Duration of GAHT (years) | 6.0 (2.8) | 7.8 (2.6) | 6.1 (1.7) | 6.3 (3.4) |
| Still using GHS | 3 (6.8%) | 1 (16.7%) | 1 (2.3%) | 7 (35%) |
| GAHT medication | Testosterone esters im: 41 (87.2%)  Testosterone undecanoate im: 5 (10.6%)  Testosterone gel: 1 (2.1%) | Estradiol valerate: 5 (83.3%)  Estradiol gel: 1  (16.7%) | Testosterone esters im: 39 (88.6%)  Testosterone undecanoate im: 4 (9.1%%)  Testosterone gel: 1 (2.3%) | Estradiol valerate: 19 (95%)  Estradiol gel: 1  (5%) |
| Estradiol  (M: 8.0-42.0 ng/L  F: 50-400 ng/L, depending on menstrual phase) | 33.0 (23.5) | 68.0 (6.0) | 29.5 (16.0) | 67.8 (56.4) |
| Total testosterone  (M: 267-929 ng/dL  F: 11-59 ng/dL) | 380.4 (399.0) | 16.7 (10.3) | 588.7 (493.9) | 18.3 (7.5) |
| Free testosterone  (M: 6-25 ng/dL  F: 0.02-0.64 ng/dL) | 7.8 (5.4) | 0.0 (0.2) | 12.9 (9.7) | 0.2 (0.3) |
| BMI >30 kg/m^2^ | 9 (19.1%) | 1 (16.7%) | 7 (15.9%) | 3 (15.%) |
| LDL-C >116 mg/dL | 13 (27.7%) | 1 (16.7%) | 15 (34.1%) | 2 (10%) |
| HDL-C below recommended for AG | 15 (31.9%) | 1 (16.7%) | 7 (15.9%) | 6 (30%) |
| Never smoker | 28 (59.6%) | 1 (16.7%) | 20 (45.5%) | 11 (55%) |
| Former smoker | 7 (14.9%) | 4 (66.7%) | 3 (6.8%) | 1 (5%) |
| Current smoker | 11 (23.4%) | 1 (16.7%) | 19 (43.2%) | 6 (30%) |
| Regular alcohol drinkers | 16 (34%) | 3 (50%) | 13 (29.5%) | 4 (20%) |
| Insufficient physical activity levels (<150 min/week)** | 2/41 (4.9%) | 1/4 (25%) | 0/33 (0%) | 0/11 (0%) |

All comparison between trans men and trans women of the two cohorts are not significant.

*4 trans men in the cohort declining the cardiological assessment did not receive any GHS.

**Not all participants agreed to wear the device for the evaluation of physical activity.

**Supplementary table 2.** Definition of cardiovascular risk factors according to guidelines.

| **Cardiovascular risk factor** | **Definition** |
| --- | --- |
| Hypertension | Systolic blood pressure >140 mmHg and/or diastolic blood pressure >90 mmHg. Systolic blood pressures between 120 and 139 mmHg and diastolic blood pressures between 70 and 89 mmHg are considered elevated [22] |
| Obesity | Class I for a BMI of 30–34.9 kg/m², class II for a BMI of 35–39.9 kg/m², and class III for a BMI ≥40 kg/m² [23] |
| Increased waist circumference | >102 cm in men  >88 cm in women [24] |
| Dyslipidemia | Triglycerides >150 mg/dL, total-C >190 mg/dL, HDL-C <40 mg/dL in men and <50 mg/dL in women, LDL-C >100 mg/dL in individuals at intermediate cardiovascular risk, or >116 mg/dL in those at low risk [24] |
| Impaired glucose tolerance | Fasting glucose >100 mg/dL [25] |
| Smoking status | Never smoker, former smoker, current smoker [26] |
| Alcohol consumption | Non-drinker if alcohol consumption <1 unit/week or drinker for alcohol consumption ≥1 unit/week.  Low-risk (1–10 units/week for men, 1–7 for women), moderate-risk (11–21 for men, 8–14 for women), high-risk (>21 for men, >14 for women) [27] |
| Physical activity | Insufficient (<150 minutes of moderate-intensity activity per week), recommended (between 150–300 minutes of moderate-intensity activity per week) and high ( >300 minutes of moderate-intensity activity per week) [28] |
